# TEACHING THE CORNELL-PENN INTERVIEW FOR DECISIONAL ABILITIES (IDA) TO ENHANCE DECISIONAL ABILITY ASSESSMENTS

**DOI:** 10.1093/geroni/igae098.2615

**Published:** 2024-12-31

**Authors:** David Hancock, Bess White, David Lachs, Jason Karlawish, Mark Lachs

**Affiliations:** Weill Cornell Medicine, New York, New York, United States; Weill Cornell Medicine, New York, New York, United States; Weill Cornell Medicine, New York, New York, United States; University of Pennsylvania, Philadelphia, Pennsylvania, United States; Weill Cornell Medicine, New York, New York, United States

## Abstract

Conventional approaches used by non-medical professionals to determine an individual’s ability to make decisions usually focus on cognitive indicators rather than the ability to reason around the particular risk at hand. The Cornell-Penn Interview for Decisional Abilities (IDA) is a novel, semi-structured interview tool that determines an individual’s ability to make decisions around a specific risk they are facing, such as the choice to remain in an abusive situation or refuse medical care. The IDA tool addresses a significant gap in the field of gerontology and promotes an individual’s right to self-determination while ensuring their safety and well-being. This study further validates the IDA by demonstrating the acceptability and efficacy of the educational program developed to teach the IDA to Adult Protective Services (APS) workers. The effectiveness of the IDA training was evaluated through feedback from 349 practitioners who completed the IDA training program. Participants rated the value of the learning experience highly, with an average score of 4.26 out of 5. Additionally, sentiment analysis of free response text using three different dictionaries (AFINN, BING, NRC) revealed overwhelmingly positive feedback, with words like “helpful,” “confident,” and “comfortable” frequently mentioned. The IDA represents a significant advancement in the assessment of decisional ability in the field of gerontology. This study proves that the IDA educational program effectively instructs APS professionals in using the IDA successfully as a rigorous, evidence-based approach to understand an APS client’s ability to make decisions around risks they are facing.

